# Detection and Identification of Pesticides in Fruits Coupling to an Au–Au Nanorod Array SERS Substrate and RF-1D-CNN Model Analysis

**DOI:** 10.3390/nano14080717

**Published:** 2024-04-19

**Authors:** Pengxing Sha, Chushu Zhu, Tianran Wang, Peitao Dong, Xuezhong Wu

**Affiliations:** Colleage of Intelligence Science and Technology, National University of Defense Technology, Changsha 410073, China

**Keywords:** surface-enhanced Raman scattering, Au–Au nanorod array, pesticide, galvanic cell reaction, 1D-CNN model analysis

## Abstract

In this research, a method was developed for fabricating Au–Au nanorod array substrates through the deposition of large-area Au nanostructures on an Au nanorod array using a galvanic cell reaction. The incorporation of a granular structure enhanced both the number and intensity of surface-enhanced Raman scattering (SERS) hot spots on the substrate, thereby elevating the SERS performance beyond that of substrates composed solely of an Au nanorod. Calculations using the finite difference time domain method confirmed the generation of a strong electromagnetic field around the nanoparticles. Motivated by the electromotive force, Au ions in the chloroauric acid solution were reduced to form nanostructures on the nanorod array. The size and distribution density of these granular nanostructures could be modulated by varying the reaction time and the concentration of chloroauric acid. The resulting Au–Au nanorod array substrate exhibited an active, uniform, and reproducible SERS effect. With 1,2-bis(4-pyridyl)ethylene as the probe molecule, the detection sensitivity of the Au–Au nanorod array substrate was enhanced to 10^−11^ M, improving by five orders of magnitude over the substrate consisting only of an Au nanorod array. For a practical application, this substrate was utilized for the detection of pesticides, including thiram, thiabendazole, carbendazim, and phosmet, within the concentration range of 10^−4^ to 5 × 10^−7^ M. An analytical model combining a random forest and a one-dimensional convolutional neural network, referring to the important variable-one-dimensional convolutional neural network model, was developed for the precise identification of thiram. This approach demonstrated significant potential for biochemical sensing and rapid on-site identification.

## 1. Introduction

Surface-enhanced Raman spectroscopy (SERS) is regarded as a promising sensing tool due to its detailed fingerprint information. This technique has found a widespread application across various domains, such as food safety [1], cancer diagnostics [2,3], drug testing [4], healthcare [5], and environmental monitoring [6]. The performance of SERS significantly depends on the sensitivity and signal reproducibility of the substrates. Detection at the single-molecule level is achievable when a molecule is positioned at highly sensitive SERS sites, often referred to as ‘hottest spots’, typically found within nano-scale gaps (usually several nanometers) between adjacent silver (Ag) or gold (Au) nanostructures [7]. Consequently, two types of substrates, solid and colloidal, made from noble metals, particularly Ag or Au, have attracted significant interest.

Colloidal substrates, despite their use, are limited due to stability and reproducibility issues, leading to a preference for solid substrates in the mass production of SERS-based sensors. These solid substrates allow for controlled structure parameters and gaps [8]. Various methods to fabricate high-performance SERS solid-state substrates have been proposed, including porous anodic alumina oxide [9], nano-sphere lithography [10], and electron beam lithography [11]. Nonetheless, these methods are either complex or costly. Oblique angle deposition (OAD), a physical vapor deposition technique [12], utilizes the self-shadowing effect for the maskless fabrication of nanostructures, offering advantages such as precise control over geometric parameters, simplicity in fabrication, good sensitivity, and high reproducibility. Recently, OAD has been utilized to fabricate an Ag nanorod array (NRA) [13,14,15,16] and Au NRA [17] that serve as SERS substrates. However, the AgNRA substrate suffers from limitations, including poor stability and biocompatibility, whereas the AuNRA array substrate, possessing good stability and biocompatibility, is limited due to its relatively low sensitivity. Therefore, a new SERS substrate possessing good stability, biocompatibility, and high sensitivity is necessary. Recent studies have focused on the development of composite substrates by grafting the metal nanoparticles (NPs) on the solid substrate to further enhance SERS performance [8], such as a composite SERS substrate prepared by grafting the Au@Ag core-shell NPs on an Au film over nanoparticles (Au FON) [18] or a composite SERS substrate prepared by grafting the Au@Ag core-shell NPs on an Ag NRA [8]. However, the process of composite substrates’ fabrication involves the synthesis of NPs, and the binding between the solid substrate and the NPs remains complex. There is a pressing need for simpler methods to prepare a composite SERS substrate. SERS substrates prepared via galvanic cell reactions, which facilitate the formation of nanostructures with numerous hot spots in a straightforward manner, have been explored for this purpose [19,20,21]. In light of this, a composite substrate featuring a wide area of granular structure was created on an AuNRA substrate through galvanic cell reactions, aiming to boost its SERS efficiency.

The deployment of SERS technology to address real-world issues has been extensively investigated. Thiram, carbendazim (CBZ), and thiabendazole (TBZ) are key components in many fungicides, while phosmet serves as an effective broad-spectrum organophosphorus insecticide. The improper use of phosmet has resulted in environmental contamination and food safety concerns. The presence of pesticide residues in agricultural produce and water systems poses risks to wildlife, domestic animals, and humans. Thus, the highly sensitive detection and identification of these insecticides are crucial. With benefits including simple pretreatment, high sensitivity, and rapid response, SERS is a promising approach to pesticide residues’ detection. Researchers are currently refining the structure of SERS substrates to boost detection sensitivity, such as Au@Ag NPs [22], a hollow silica microspheres@Au NPs composite [23], and a titanium carbide MXene/Ag nanostars composite [24]. However, these SERS sensors can only detect a single pesticide residue; the selectivity of SERS to detect each of the specific pesticides in a mixture simultaneously when multiple pesticides are present in a sample is a major challenge.

Machine learning algorithms, such as principal component analysis (PCA) [25,26,27,28], k-nearest neighbor (KNN) methods [27,28], random forest (RF) methods [29], and support vector machines (SVMs) [29,30,31], are being increasingly applied in Raman spectroscopy analyses to elucidate the molecular structural information provided via SERS spectra [32]. Despite being powerful, they still encounter problems when applied to complex-system detection. More powerful algorithms for Raman spectral analysis are needed.

In this study, a simple approach based on galvanic cell reactions was developed to improve the SERS performance of AuNRA substrates by depositing large-area Au nanostructures on the surface of the AuNRA. Various pesticides, such as thiram, TBZ, CBZ, and phosmet, were detected using the prepared Au–Au NRA substrate, and spectral datasets were established. To meet the requirements of the rapid on-site identification of pesticide residues, a combined RF and one-dimensional convolutional neural network (1D-CNN) named the important variable-one-dimensional convolutional neural network model (RF-1D-CNN) was developed. Even for a variety of pesticide residues, RF-1D-CNN could accurately identify the presence of thiram residues. The actual detection conditions were simulated. Thiram residue was detected at a concentration of 24 ng/cm^2^ on cucumber surfaces.

## 2. Materials and Methods

### 2.1. Materials and Chemicals

Chloroauric acid (HAuCl_4_·4H_2_O, 99.99%) was procured from Aladdin (Shanghai, China). Thiabendazole (TBZ) and a carbendazim standard solution (CBZ) were acquired from Macklin (Shanghai, China). Phosmet and thiram were obtained from Aladdin, and 1,2-bis (4-pyridyl) ethylene (BPE) was sourced from Sigma-Aldrich (St. Louis, MO, USA). Si wafers served as the substrate. Gold (Au) pellets with a purity of 99.99% and chromium (Cr) pellets with a purity of 99.999% were purchased from Jinyu Aochen (Beijing, China). Ethanol (C_2_H_5_OH), sulfuric acid (H_2_SO_4_), and a hydrogen peroxide solution (H_2_O_2_) were obtained from Sinopharm (Shanghai, China). All chemicals were utilized as received without purification. Milli-Q water (resistance rate ≥ 18.2 MΩ·cm) was utilized as the water sample.

### 2.2. Apparatus

The AuNRA substrate was prepared on a Si wafer using an e-beam evaporator (ZZS500, Nanguang, Chengdu, China). The morphologies of the Au–Au NRA were characterized using scanning electron microscopy (SU-8010, Tokyo, Japan) with an accelerating voltage of 3.0 kV. Raman spectra were collected with a portable Raman system equipped with a 785 nm excitation source (BWS415–785S, from B&W, TeK, Plainsboro Township, NJ, USA).

### 2.3. FDTD Model

The simulation model of the Au–Au NRA composite structure substrate is depicted in Figure S1. To streamline the calculation and refine the simulation outcomes, the two-dimensional simulation mode was opted for during model execution. The Y axis represented the direction normal to the substrate, while the X axis denoted the growth direction along the nanorod (NR) and the axis perpendicular to the Z and Y axes. The size of the Si wafer in the model measured 3000 × 1000 nm. In accordance with the experimental parameters, Ti (20 nm) and Au layers (100 nm) were deposited on the Si wafer surface. For simplification and a reduced calculation time, only three rows and columns of the AuNRA were utilized. The diameter, length, and tilt angle of AuNRs were set to 80 nm, 800 nm, and 71°, respectively. The distances between two NRs along the X and Z axes were set to 550 nm and 150 nm, respectively. The parameters of AuNRA utilized in the FDTD simulation analysis were derived from actual experimental findings. To investigate the enhancement effect of the NRA composite structure and streamline the model, Au NPs were positioned solely at the top of the NRs and between adjacent NRs based on the AuNRA structure. The diameter of the Au NPs was defined as 40 nm. A plane wave with a wavelength of 785 nm served as the laser source, and it was perpendicular to the substrate. The EM field distribution on the XY plane of the model was observed with a grid division accuracy of 0.1 × 0.1 nm.

### 2.4. Preparation of the Au–Au NRA Substrate

The composite structure was derived from the AuNRA array substrate, serving as a supporting structure. Through the process of galvanic-cell-reaction-driven deposition, a substantial area of Au nano-granular structure was formed on the surface of NRA. Typically, the preparation of the composite structure involved two steps. Initially, an Au NRA was generated using the OAD technique with an e-beam evaporator. A 20-nm-thick Ti layer followed by a 100-nm-thick Au layer was deposited on a Si wafer cleaned with piranha solution in the specified sequence. Subsequently, the wafer was tilted to an angle of 86°, determined according to the angle between the normal direction of the Si wafer and the direction of the evaporation source. The film thicknesses, as measured via quartz crystal microbalance (QCM), were adjusted to regulate the length of the AuNRs to 800 nm, with deposition rates set to 3.5 Å/s. The prepared AuNRA substrate was connected to copper foil via wires and secured in the reaction box. A 2.5 mM HauCl_4_ aqueous solution served as the electrolyte in the experiment, transferred to a galvanic cell. A rotor was introduced into the reaction box, and the speed was adjusted to 650 rpm to ensure the uniformity of the reaction solution. Following deposition for 10 min at room temperature, the products were retrieved, cleansed with distilled water (DIW) multiple times, and dried with high-purity flowing nitrogen.

### 2.5. Raman Measurements

Raman spectra were obtained using a BWS465–785H Raman microscope (B&W, TeK, Plainsboro Township, NJ, USA) equipped with a 785 nm excitation laser, with spectra collected over 10 s integration times and a laser power of approximately 30 mW. All SERS spectra used for curve depiction were baseline-corrected and averaged over three randomly selected spots of a given substrate. The SERS enhancement ability of the AuNRA and Au–Au NRA substrate was evaluated using BPE. For the detection of TBZ, CBZ, phosmet, and thiram, the Au–Au NRA substrates were immersed in the corresponding pesticide solution for 3 h.

In this study, binary (thiram and TBZ) and ternary (thiram, TBZ, and CBZ) mixtures were prepared by mixing different volumes of pesticides, with each pesticide solution having a concentration of 10^−5^ M. In the binary system, the mixing volume ratios (thiram:TBZ) were 5%:95% and 10%:90%. In the ternary system, the mixing volume ratios (thiram:TBZ:CBZ) were 5%:47.5%:47.5% and 10%:45%:45%, respectively.

To simulate an actual sample detection environment, thiram ethanol solution was dropped onto the surface of a cucumber and tested. The cucumber, obtained from a local supermarket, was ultrasonically washed multiple times with DIW, then cleaned with ethanol to eliminate contaminants and organic matter, and dried with nitrogen. Approximately 1 cm × 1 cm squares of cucumber skin were excised with a clean knife. A total of 1 µL of 10^−4^ M thiram ethanol solution was evenly spread on the cucumber skin and air-dried. Subsequently, the thiram on the cucumber skin was eluted using filter paper moistened with ethanol, with the filter paper placed in 500 µL of ethanol solution. The prepared Au–Au NRA substrate was immersed in the solution for 3 h.

All the spectra were obtained using a Raman microscope after the sample solution was air-dried.

### 2.6. Data Processing and Division

In this experiment, the Raman spectra of four pesticides at various concentrations were collected. The concentrations of each pesticide were 10^−4^ M, 5 × 10^−5^ M, 10^−5^ M, 5 × 10^−6^ M, 10^−6^ M, and 5 × 10^−7^ M. Forty spectra were randomly acquired for each concentration of each pesticide. The Raman intensity corresponding to Raman shifts across the entire spectrum from 400 cm^−1^ to 1800 cm^−1^ was initially recorded. Although the obtained Raman spectrum data constituted a small sample dataset, establishing a stable and reliable classification model typically necessitates a large volume of data. Hence, the sample data were extended to enhance data diversity using three data augmentation methods: (a) shifting the spectrum randomly to the left or right within a small range; (b) introducing random noise to the spectrum; and (c) forming linear combinations of spectra belonging to the same substance at the same concentration with the sum of corresponding coefficients equal to 1. Following data expansion, 1440 spectra were derived from standard solutions for each pesticide. For model construction, 1300 data points were randomly selected. Eighty percent of the data served as the training set, while twenty percent constituted the validation set.

Thiram spectra were designated as the positive sample, while the Raman spectra of the other three pesticides (TBZ, CBZ, and phosmet), along with the substrate spectra containing only ethanol, were considered the negative sample. Positive samples were labeled 1, whereas negative samples were labeled 0. Consequently, the training set for the pesticide classification model comprised 2080 spectral data points, while the validation set contained 520 spectral data points.

Prior to data input, the RF model was established and optimized. Subsequently, the training set was examined to identify and extract the important variables of the spectrum. The optimized RF model consisted of 50 decision trees with an eigenvalue of 100. The important variables were predominantly concentrated near the Raman shifts of 1000 cm^−1^ and 1400 cm^−1^. To reduce data dimensionality and ensure model generalizability, the first 450 most important variables were utilized as input for the CNN model. Following variable selection in the RF model, the input data were reduced from 900 to 450 dimensions.

### 2.7. RF-1D-CNN Model Construction

The Raman spectrum is a one-dimensional signal. The RF-1D-CNN model designed in this study contains four convolution layers, two pooling layers, and three fully connected layers. One batch of normalization layers was added after each layer, and a dropout layer with a random inactivation ratio *p* = 0.5 was applied after the second fully connected layer to prevent overfitting. The hyperparameters (including batch_size, learning rate, random inactivation ratio, weight_decay, and momentum) of our RF-1D-CNN model were optimized with a random search. The sizes of the RF-1D-CNN kernels were 32, 64, 128, and 64. Max pooling was used in the pooling layer. The SoftMax function was used in the output layer of the qualitative model. Each Raman spectrum was input into the neural network in the form of a one-dimensional tensor.

In the experiment, a dataset comprising 2600 spectra was established to train and validate the models. This dataset consisted of 1300 positive SERS spectra (the SERS spectra of thiram) and 1300 negative SERS spectra (the SERS spectra of the other three pesticides and the substrate, totaling 325 spectra for each). In the dataset, twenty percent of the data was selected randomly as the validation set, while the remaining eighty percent of the data served as the training set, and the CrossEntropyLoss of the RF-1D-CNN model on the validation set was used to evaluate the performance of the hyperparameter configurations. K-fold cross-validation was utilized to estimate the stability of the RF-1D-CNN model, wherein the value K was set to 5.

To evaluate the recognition accuracy of the RF-1D-CNN model, the SVM [31], RF [29], and KNN [33] models were used for comparison. The parameters of the three models were optimized. For the establishment of all the models, the spectra obtained were baseline-corrected using the airPLS [34] algorithm. The SVM and KNN models also used normalization pre-processing before data input.

### 2.8. Identification and Evaluation of Models

The metrics of accuracy, sensitivity, and specificity were calculated as follows:

(1) $$accuracy=\frac{TP+TN}{TP+TN+FP+FN}$$

(2) $$sensitivity=\frac{TP}{TP+FN}$$

(3) $$specificity=\frac{TN}{TN+FP}$$

where *TP*, *TN*, *FP*, and *FN* represent true positive, true negative, false positive, and false negative, respectively.

## 3. Results and Discussions

### 3.1. Synthesis and Morphological Characterization of the Au–Au NRA Composite Structure

**Figure 1:**
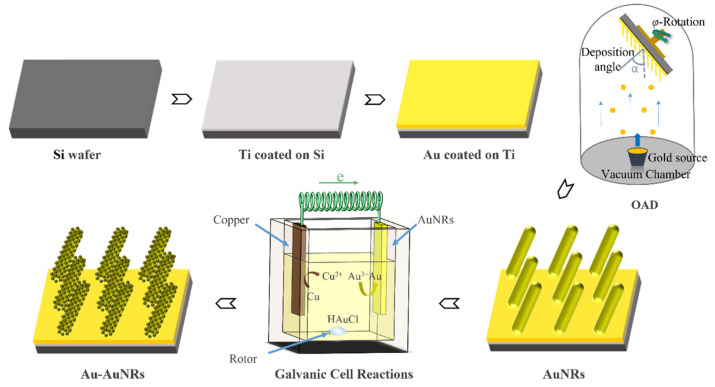
Schematic of the fabrication process of the Au–Au NRA substrate.

First, an Au NRA was produced using the OAD technique according to our previous studies [17]. The prepared AuNRA substrate was subsequently used as the cathode and immersed in a chloroauric acid (HauCl_4_) solution together with copper foil as the anode. A complete galvanic cell was formed by connecting the AuNRA substrate and copper foil with wires. Under the action of galvanic cell reactions, a large area of an Au nano-granular structure was formed on the surface of the NRs, which further increased the number of SERS hot spots and improved the SERS performance of the substrate. The fabrication procedure is shown in Figure 1.

To elucidate the growth mechanism of the Au–Au NRA substrate, the impact of HauCl_4_ concentration on the formation of this composite substrate was initially investigated. As depicted in Figure S2, a granular structure of a certain extent formed on the surfaces of NRs when the HauCl_4_ concentration was 1 mM, albeit with a small particle size. With the HauCl_4_ concentration increased from 1 to 2.5 mM, the granular structure continued to expand within the same reaction time, accompanied by narrowing gaps between neighboring NRs. Upon further increasing the HauCl_4_ concentration to 5 mM, the granular structures in the vertical direction of the NRs nearly coalesced, leading to the crosslinking of NRs in the AuNRA substrate.

Subsequently, the effect of different durations of Au deposition on morphology was explored, as shown in Figure S3. The morphology of the Au–Au NRA substrate could be effectively tailored by varying the deposition time. Initially, only a small quantity of Au NPs was assembled on the surface of the AuNRA at the onset of the reaction. However, with a prolonged deposition time, the number and size of Au NPs gradually increased, eventually coating the entire surface of the NRA. Concurrently, the spacing between adjacent NRs progressively diminished. Upon extending the deposition time to 15–20 min, the NRs in the AuNRA substrate also approached crosslinking.

Based on the aforementioned results, it can be inferred that the morphology of the Au–Au NRA substrate can be regulated by controlling the amount of HauCl_4_ and the reaction time of the galvanic cell reaction growth.

**Figure 2:**
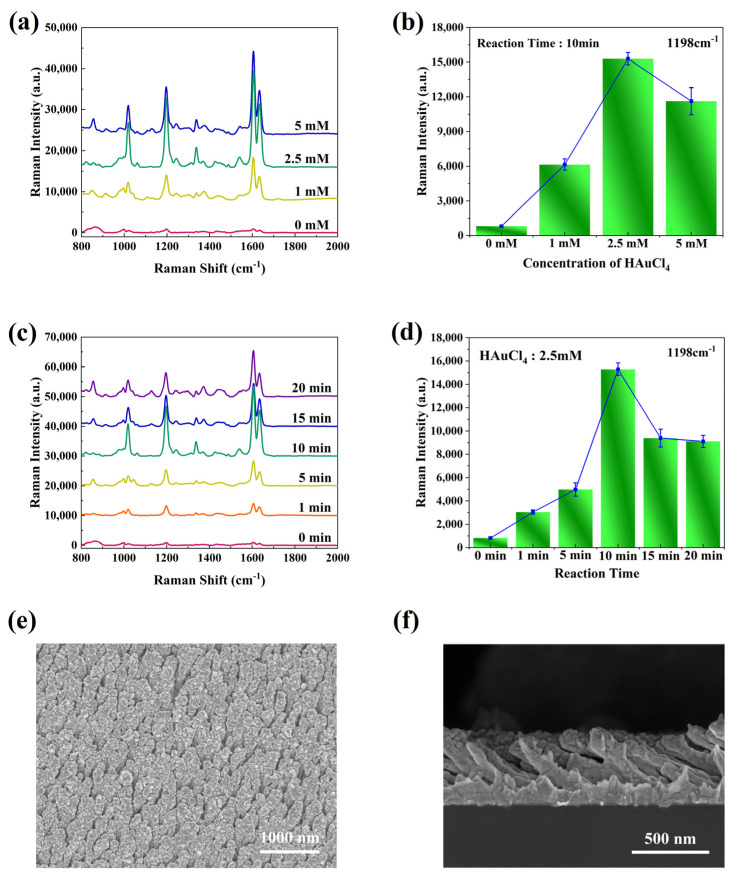
SERS spectra of BPE and Raman intensity distribution at the Raman shift of 1198 cm^−1^ collected on an Au–Au NRA composite structure with (**a**,**b**) different concentrations of HauCl_4_, (**c**,**d**) different durations of Au deposition, (**e**,**f**) Au–Au NRA observed under a top view and a cross-sectional view after reacting with 2.5 mM of HauCl_4_ for 10 min. Data points represent the mean of five repetitions with error bars (standard deviation).

The size and spacing of the NRA structure profoundly influenced the SERS performance of the NRA substrate. Thus, the optimal amount of HauCl_4_ and the reaction time were investigated by testing the SERS activity of the as-fabricated Au–Au NRA substrate using 10^−6^ M BPE as probe molecules under various experimental conditions, and the results are shown in Figure 2.

BPE molecules exhibit SERS peaks primarily at the vibration bands of 1198 cm^−1^, 1605 cm^−1^, and 1636 cm^−1^. These bands correspond to the C=C stretching mode, aromatic ring stretching, and in-plane ring mode, respectively, consistent with the literature [35]. The composite structure substrate demonstrated higher SERS activity compared to the pure AuNRA substrate, suggesting that the presence of large-area granular structures can enhance the SERS activity of the substrate. Figure 2a depicts the effects of different HauCl_4_ concentrations on the substrate performance. With a fixed reaction time of 10 min, the SERS signal intensity notably improved as the concentration of HauCl_4_ increased from 1 mM to 2.5 mM. However, the signal intensity slightly decreased when the concentration of HauCl_4_ was further increased to 5 mM. The average intensities of spectra measured at the vibration bands of 1198 cm^−1^ are presented in Figure 2b. The Au–Au NRA substrate achieved the maximum SERS enhancement when the HauCl_4_ concentration was 2.5 mM. Therefore, a HauCl_4_ solution with a concentration of 2.5 mM was selected as the electrolyte. As illustrated in Figure 2c, the SERS signal intensity of the Au–Au NRA substrate initially increased and then decreased with the increase in the reaction time. Figure 2d displays the average intensity of the spectral peak measured at the Raman shift of 1198 cm^−1^, indicating that the Au–Au NRA substrate obtained when the reaction time was 10 min exhibited the highest SERS activity. In summary, the large-area NP structure formed on the surface of the NRs through galvanic-cell-reaction-driven deposition effectively improved the SERS activity of the substrate. Additionally, the Au–Au NRA substrate, prepared by reacting in a 2.5 mM HauCl_4_ solution for 10 min, served as the optimal SERS substrate. SEM images of the top and cross-section views of the Au–Au NRA substrate prepared under optimized conditions are depicted in Figure 2e and 2f, respectively, and a high-magnification SEM image of the top view is depicted in Figure S4, revealing evenly distributed NP structures on every NR. Moreover, the Au–Au NRA composite substrate exhibited a periodic array structure and maintained the integrity of the NRA substrate.

### 3.2. SERS Performance Characterization

**Figure 3:**
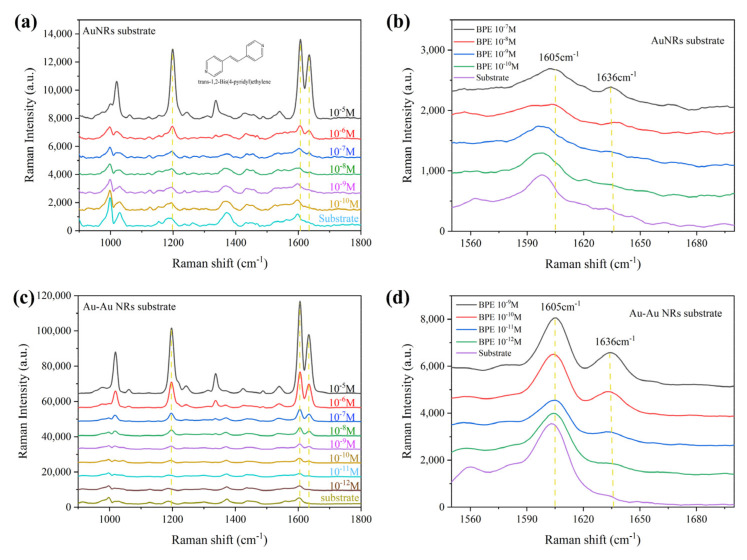
SERS spectra of BPE and a partial enlargement from 1560 cm^−1^ to 1680 cm^−1^ detected for (**a**,**b**) pure AuNRA and (**c**,**d**) Au–Au NRA.

To further compare the SERS enhancement ability of the AuNRA and the optimal Au–Au NRA substrate, Raman spectra were acquired using BPE as the probe molecule. A series of concentrations of BPE ethanol solution ranging from 10^−5^ M to 10^−12^ M was prepared. The BPE solution (2 µL) was dropped on the two substrates. Then, the substrates were air-dried prior to measurement. The average SERS spectra of BPE detected on the AuNRA and Au–Au NRA substrates are shown in Figure 3. All the SERS spectra showed the characteristic peaks of BPE. Histograms of Raman intensity at 1636 cm^−1^ detected for pure AuNRA and Au–Au NRA are shown in Figure S5a and S5b, respectively; significant difference analysis indicates that the LOD of the AuNRA substrate was 10^−6^ M, and the LOD of the Au–Au NRA substrate was 10^−11^ M, five orders of magnitude greater than that the AuNRA substrate. Furthermore, using GraphPad Prism 8 software, the standard curve of Raman intensity vs. the BPE concentration logarithm was generated; it was fitted to a sigmoidal 4 PL model, and the non-linear regression analysis yielded an R^2^ value of 0.9963.

The uniformity of the SERS signal for the optimal Au–Au NRA substrate was evaluated by recording the signal intensity distribution at 1198 cm^−1^ for 10^−6^ M, 10^−9^ M, and 10^−10^ M BPE, respectively, from 30 sites on the surface of the Au–Au NRA substrate. The qualitative study in the histogram of Figures S6–S8 indicates nearly similar intensities of the 1198 cm^−1^ Raman peak with a low relative standard deviation of approximately 6.1%, 8.2%, and 5.7%. The as-prepared Au–Au NRA substrate had relatively good uniformity.

All in all, the Au–Au NRA substrate fabricated in this study offers advantages, including high sensitivity, good stability, and simplicity in fabrication. The Au–Au NRA substrate exhibits a sensitivity comparable to that of the AgNRA substrate [36], and it possesses good stability and biocompatibility, which the AgNRA substrate lacks. Additionally, the fabrication process of our SERS substrate using an OAD process with galvanic cell reactions is fairly simple; the deposition of large-area Au nanostructures on AuNRA only requires 10 min, far shorter than that required for the traditional composite substrate fabrication process [8,18].

### 3.3. FDTD Calculation

**Figure 4:**
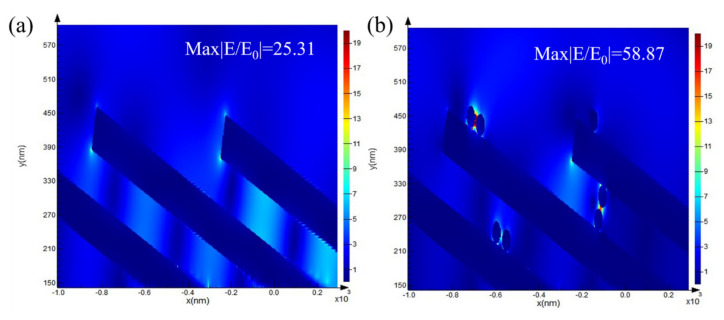
Results of the FDTD method for the NRA array substrate: (**a**) pure AuNRA and (**b**) the Au–Au NRA composite structure.

The simulation results of the electromagnetic (EM) field distributions of the different models are shown in Figure 4. Since the prepared AuNRA has a hydrophobic structure, deposition via galvanic cell reaction mainly occurs within a range near the top of the NRs. Therefore, in the analysis of the simulation results, the EM field distribution near the top of the NRs was studied. The Au–Au NRA composite structure (Figure 4b) contains more hot spots than the original AuNRA (Figure 4a) due to the existence of a granular structure. This increase is mainly due to the hot spots that formed between the NPs and between the NPs and the NRs. In addition to the increase in the hot spot density, the Au–Au NRA composite structure also exhibited a higher electromagnetic intensity. An increase in the SERS hotspot density and intensity improved the SERS effect compared with that of the pure AuNRA substrate.

### 3.4. SERS Detection of Pesticides

**Figure 5:**
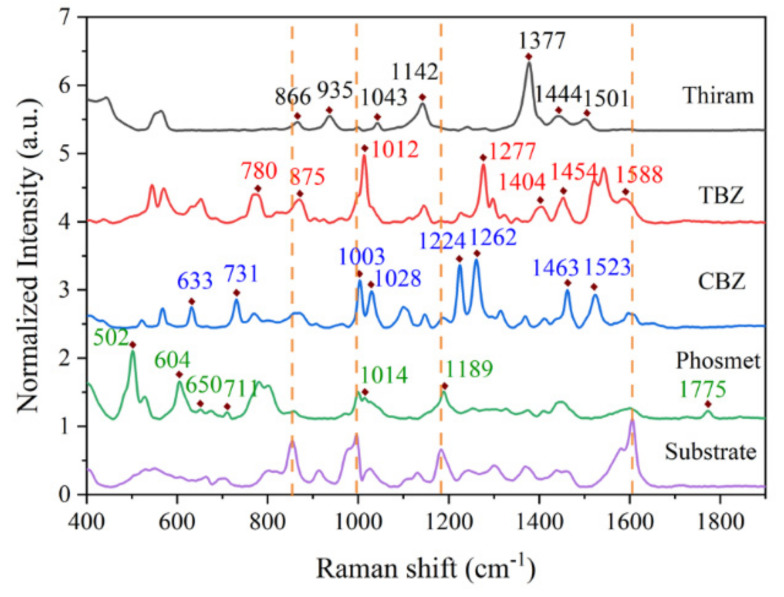
Normalized spectra of different analytes (10^−5^ M) and the control.

The optimal Au–Au NRA was used to detect four different pesticides at various concentrations, and a spectral dataset was simultaneously established. Figure S9 shows the chemical structures of the four different pesticides, and Figure 5 shows the spectrum for each of the analytes against a blank sample. The characteristic peaks for each of the analytes can be clearly identified and are near matches to known band positions, and the assignments of the SERS bands of thiram [37], TBZ [38,39], CBZ [40], and phosmet [41] are shown in Tables S1, S2, S3, and S4, respectively. The specific detection of four pesticides was achieved using the prepared composite structure substrate. To ensure the reproducibility of our SERS substrate, five batches of substrates in parallel were utilized to detect the above four different pesticides, respectively, and eight different laser spots on each substrate were chosen to yield SERS signals. The relative standard deviation (RSD) values of the corresponding SERS intensities of thiram, TBZ, CBZ, and phosmet were 7.2% (Figure S6), 6.7% (Figure S7), 6.7% (Figure S8), and 7.9% (Figure S9), respectively, revealing that the prepared Au–Au NRA substrate achieved high reliability and reproducibility for pesticide detection. These results indicated the significant potential of the Au–Au NRA substrate in the field of biochemical testing.

### 3.5. Identification of Pesticides Using RF-1D-CNN

To achieve the requirements for the rapid on-site identification of pesticide residues, the automatic identification of analytical spectra was performed via SERS spectral analysis methods.

**Figure 6:**
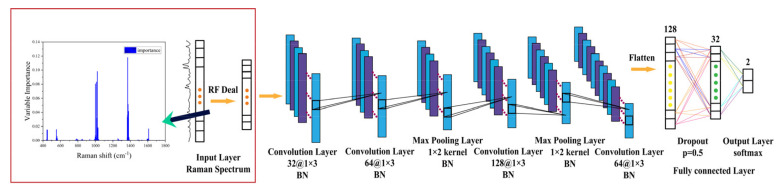
Structure of the RF-1D-CNN model based on SERS spectra.

Since the Raman spectrum is a spectral signal with a high characteristic dimension, repeated information during model training is provided due to the strong correlation between many data dimensions when the full spectral segment data are used as the input, which affects the running speed of the model. A reasonable dimensionality reduction can ensure the accuracy of the model classification and improve the training efficiency of the model. An RF model was established to analyze the training set and extract the important variables of the spectrum. A distribution diagram of the spectral variables important for the qualitative identification of the target pesticide thiram using the RF model is shown in Figure 6 (the inset image).

As a new data processing method, CNNs are typical feedforward neural networks, and they have attracted considerable attention in the field of spectral analysis. In this study, a novel RF-1D-CNN model combining an RF model and a 1D-CNN model was designed to identify pesticide residues specifically. A schematic of the RF-1D-CNN designed in this study is shown in Figure 6.

To assess the recognition accuracy of the RF-1D-CNN model, SVM [31], RF [29], and KNN [33] models were utilized for comparison. The accuracy rate (ACC), sensitivity, and specificity were employed to evaluate the identification accuracy of the various models.

Table S5 displays the recognition results using different models with the verification set. Remarkably, all models achieved 100% accuracy. This high accuracy may be attributed to the distinct molecular structures of the four pesticides, leading to significantly different Raman spectral characteristic peaks. Consequently, even simple machine learning models can readily identify the four pesticides.

By utilizing a binary mixture of thiram and TBZ or a ternary mixture of thiram, TBZ, and CBZ as the research object, the recognition of thiram using different models was investigated. Figure S10 illustrates the SERS spectrum obtained from the two mixtures, wherein the thiram content was 5%. Due to the blending of multiple pesticides, some SERS characteristic bands overlap, thereby increasing the challenge of feature extraction and spectral identification.

Different batches of substrates were used to detect the mixture. A total of 20 SERS spectra of binary and ternary mixed pesticide systems with different thiram contents were obtained randomly on the substrate surface and used as positive samples. Five samples of thiram, TBZ, CBZ, and substrate control SERS spectra were selected as negative samples. A total of 40 spectral test sets were constructed to verify and compare the performance of the models. Table S6 shows the results for the identification of thiram in the binary mixtures with different models. Table 1 shows the results for the identification of thiram in the ternary mixtures with different models.

Table 1 shows that the four models can be used to judge the presence of thiram accurately when the content of thiram in the mixed solution is high. With a decreasing thiram content, the detection performance of the SVM, RF, and KNN models decreased to some extent. However, the RF-1D-CNN model maintained 100% recognition accuracy.

The selectivity of SERS to detect the specific pesticide in the mixture of multiple pesticides present in a sample is a major challenge. Detecting multiple pesticides simultaneously from a sample mixture directly through their characteristic peaks is scarcely possible. This is probably because competitive adsorption to the SERS substrates occurs when multiple analytes are present; thus, the analyte with the lower binding affinity to the substrate cannot be easily detected [42]. The pre-separation of individual pesticides [43] can solve this problem, but this would inevitably increase the analytical time and complexity. A single aptamer-based SERS method was developed for the rapid detection of multiple pesticides, and four pesticides can be captured and detected using PCA, based on their distinct fingerprint Raman peaks [44], whereas this method has only been applied to identify multiple pesticides with similar concentrations. In this study, the combination of Au–Au NRA-based SERS technology and 1D-CNN allowed the successful identification of low-content (5%) thiram in the ternary mixture of thiram, TBZ, and CBZ, exhibiting greater stability, reliability, and accuracy than the other methods.

**Figure 7:**
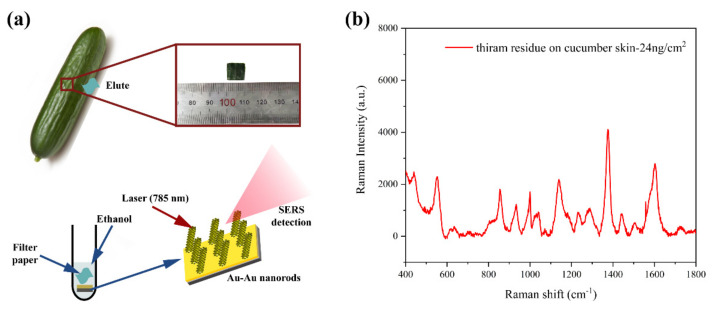
(**a**) Schematic of the detection process for cucumber peels; (**b**) SERS spectrum of cucumber peels treated with thiram.

To demonstrate the value of the combination of 1D-CNN and SERS technology in practical applications, thiram residues were simulated on cucumber skin. The thiram residue on the cucumber skin was approximately 24 ng/cm^2^. Figure 7 shows the flow chart of the simulated detection and SERS spectrum of the obtained thiram. The obtained spectra were input into the trained RF-1D-CNN model, which achieved 100% accuracy in the recognition of thiram. The experimental results showed that the combination of Au–Au NRA-based SERS technology and 1D-CNN was able to sensitively detect thiram at a level of 24 ng/cm^2^, which is lower than the previously reported thiram detection limit (38 ng/cm^2^) with Ag nanoshells [45] and much lower than the maximum permissible level of ∼2 μg/cm^2^ for fruit peels [45].

## 4. Conclusions

In this study, a method for preparing an Au–Au NRA composite structure substrate using an OAD process with galvanic cell reactions was introduced. The deposition of large-area Au nanostructures on the surface of AuNRA significantly enhanced the SERS effect of the substrate. The resulting composite structure demonstrated high sensitivity, repeatability, and uniformity. Subsequently, the SERS detection of various pesticides was conducted, and a spectral dataset was established using the prepared composite structure substrate. By integrating RF with the 1D-CNN algorithm, an RF-1D-CNN model was developed for the qualitative identification of pesticides. Compared with SVM, RF, and KNN, RF-1D-CNN exhibited the best identification performance. An actual detection environment was simulated, and the accurate identification of thiram (24 ng/cm^2^) on cucumber skin was achieved using filter paper tests, SERS detection, and RF-1D-CNN model analysis. The combination of the Au–Au NRA SERS substrate and RF-1D-CNN facilitated rapid, accurate, and automatic detection and recognition. This research holds significant value in broadening the applications of SERS technology.

## Figures and Tables

**Table 1:** Identification of thiram in mixtures of thiram, TBZ, and CBZ.

Mixture	Models	ACC (%)	Sensitivity (%)	Specificity (%)
Thiram + TBZ + CBZ ratio: 5%:47.5%:47.5%	SVM	70	100	40
	RF	50	0	100
	KNN	90	100	80
	RF-1D-CNN	100	100	100
Thiram + TBZ + CBZ ratio: 10%:45%:45%	SVM	67.5	100	30
	RF	97.5	95	100
	KNN	90	100	80
	RF-1D-CNN	100	100	100

## Data Availability

Data are available upon request from the corresponding authors.

## Supplementary Materials

The following supporting information can be downloaded at https://www.mdpi.com/article/10.3390/nano14080717/s1. Figure S1: Models of the Au–Au core-shell nanorod array (NRA) substrate. Figure S2: SEM images of the Au–Au NRA composite structure with (a) 0 mM HAuCl_4_, (b) 1 mM HAuCl_4_, (c) 2.5 mM HAuCl_4_, and (d) 5 mM HAuCl_4_. The reaction time was kept to 10 min. Figure S3: (a–f) SEM images of the Au–Au NRA composite structure after different durations of Au deposition. The concentration of HAuCl_4_ was maintained at 2.5 mM. Figure S4. Au–Au NRA observed under a high-magnification view after reacting with 2.5 mM HAuCl4 for 10 min. Figure S5. Histograms of Raman intensity at 1636 cm^−1^ detected for pure AuNRA (a) and Au–Au NRA(b). (c) Calibration curve of Raman intensity at 1636 cm^−1^ detected for Au–Au NRA. (* *p* < 0.05, ** *p* < 0.01, ns: no significance). Figure S6: The signal intensity distribution at 1198 cm^−1^ for 10^−6^ M BPE recorded from 30 sites. Figure S7: The signal intensity distribution at 1198 cm^−1^ for 10^−9^ M BPE recorded from 30 sites. Figure S8: The signal intensity distribution at 1198 cm^−1^ for 10^−10^ M BPE recorded from 30 sites. Figure S9: The chemical structures of thiram, TBZ, CBZ, and phosmet. Figure S10: The signal intensity distribution at 1377 cm^−1^ for 10^−5^ M thiram. Five batches of substrates in parallel were utilized, and eight different laser spots on each substrate were chosen to yield SERS signals. Figure S11: The signal intensity distribution at 1277 cm^−1^ for 10^−5^ M TBZ. Five batches of substrates in parallel were utilized, and eight different laser spots on each substrate were chosen to yield SERS signals. Figure S12: The signal intensity distribution at 1262 cm^−1^ for 10^−5^ M CBZ. Five batches of substrates in parallel were utilized, and eight different laser spots on each substrate were chosen to yield SERS signals. Figure S13: The signal intensity distribution at 1189 cm^−1^ for 10^−5^ M phosmet. Five batches of substrates in parallel were utilized, and eight different laser spots on each substrate were chosen to yield SERS signals. Figure S14: SERS spectrum obtained from (a) binary mixture system of thiram and TBZ and (b) ternary mixture system of thiram, TBZ, and CBZ. Table S1: Assignments of SERS bands of thiram. Table S2: Assignments of SERS bands of TBZ. Table S3: Assignments of SERS bands of CBZ. Table S4: Assignments of SERS bands of Phosmet. Table S5: Identification results for the verification set. Table S6: Identification of thiram in mixtures of thiram and TBZ.
