# Supplementary material for: Detection and Identification of Pesticides in Fruits Coupling to an Au–Au Nanorod Array SERS Substrate and RF-1D-CNN Model Analysis
Source: Nanomaterials (Basel). 2024 Apr 19;14(8):717. doi: 10.3390/nano14080717 (PMC11053652; doi:10.3390/nano14080717)
Supplement: Supplementary file 1 [file nanomaterials-14-00717-s001.zip › nanomaterials-2927890-supplementary.pdf]

Supplementary Materials

Detection and identification of pesticides in fruits coupling to an Au-Au nanorod array SERS substrate and RF-1D-CNN model analysis

Pengxing Sha [1,†], Chu-shu Zhu [1,†], Tianran Wang [1], Peitao Dong [1,*] and Xuezhong Wu [1]

[1] Colleage of Intelligence Science and Technology, National University of Defense Technology, Changsha 410073, People's Republic of China

† These authors contributed equally to this work.

* Correspondence: ptdong@nudt.edu.cn; Tel.: +86-731-87005236.

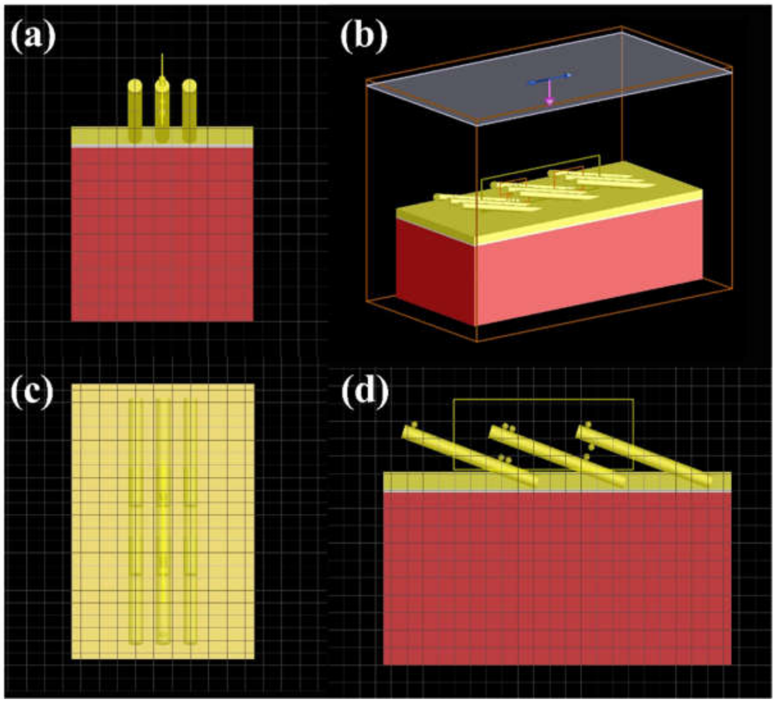

Figure S1. Models of the Au–Au core-shell nanorod array (NRA) substrate.

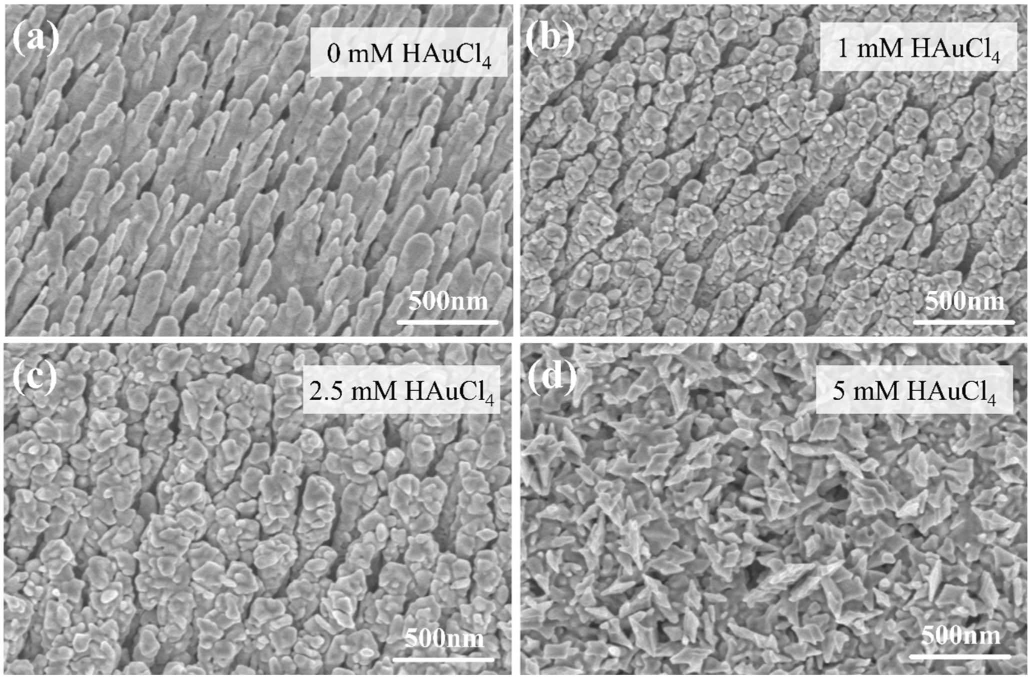

Figure S2. SEM images of the Au-Au NRA composite structure with (a) 0 mM $HAuCl_4$; (b) 1 mM $HAuCl_4$; (c) 2.5 mM $HAuCl_4$; (d) 5 mM $HAuCl_4$. The reaction time was kept at 10 min.

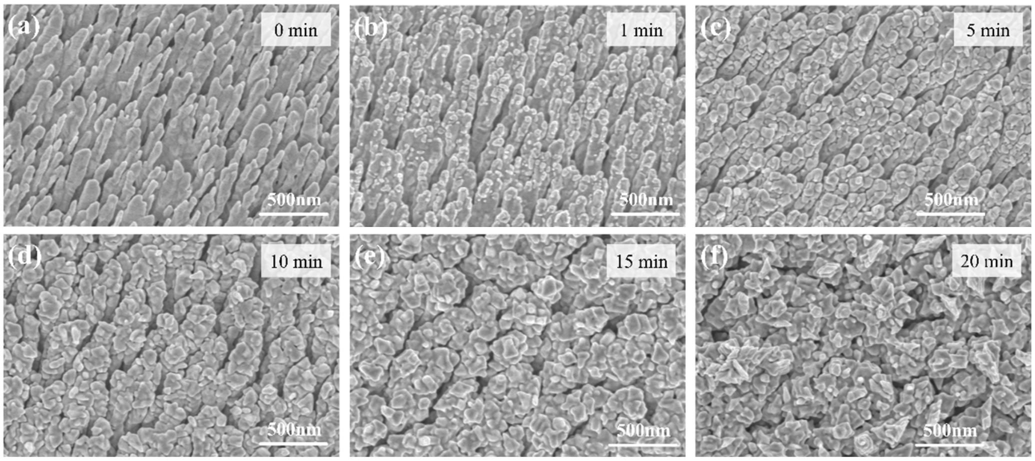

Figure S3. (a–f) SEM images of the Au-Au NRA composite structure after different durations of Au deposition. The concentration of $HAuCl_4$ was maintained at 2.5 mM.

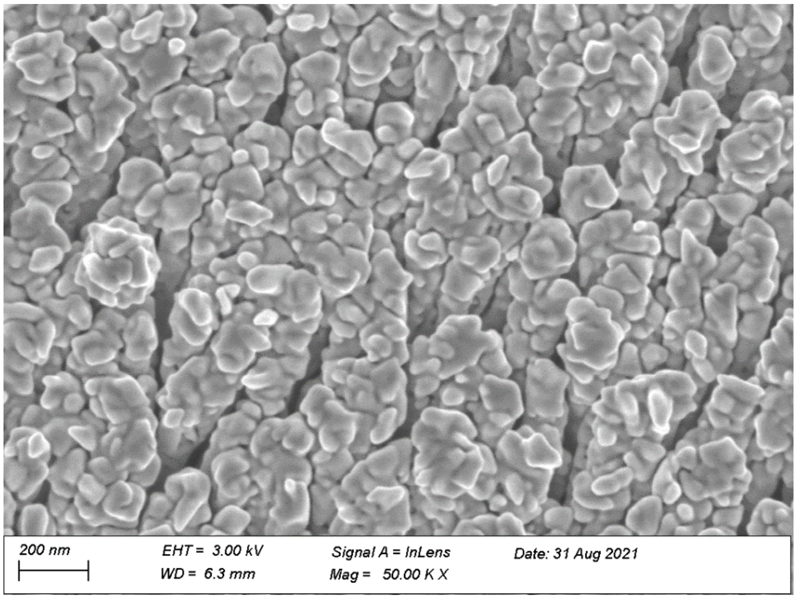

Figure S4. Au-Au NRA observed under high magnification view after reacting with 2.5 mM $HAuCl_4$ for 10 min.

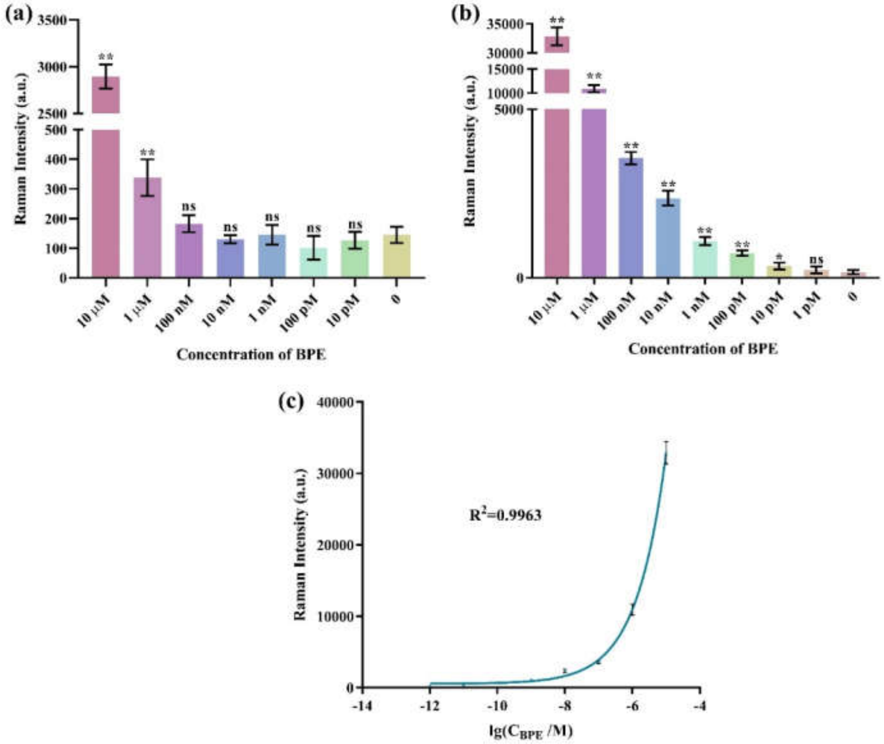

Figure S5. Histograms of Raman intensity at 1636 cm^{-1} detected for pure AuNRA (a) and Au-Au NRA(b). (c). calibration curve of Raman intensity at 1636 cm^{-1} detected for Au-Au NRA. (*$P < 0.05$, **$P < 0.01$, ns: No significance)

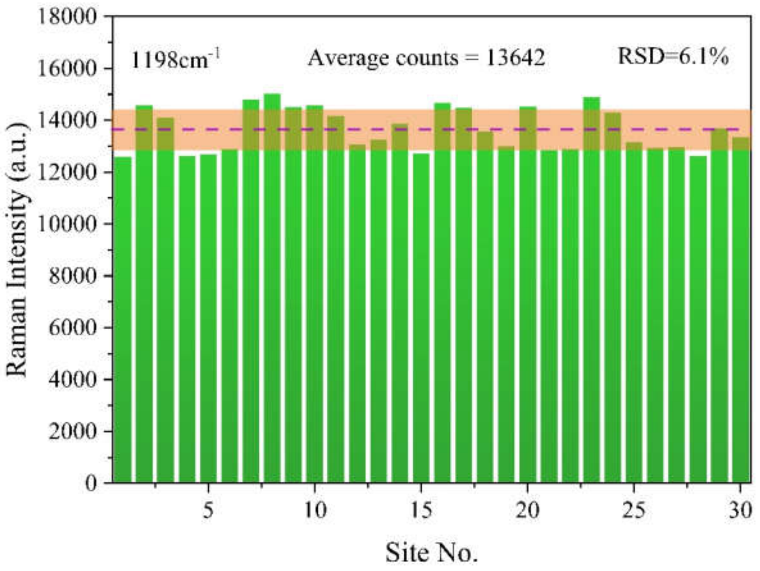

Figure S6. The signal intensity distribution at 1198 cm^{-1} for 10^{-6} M BPE recorded from 30 sites.

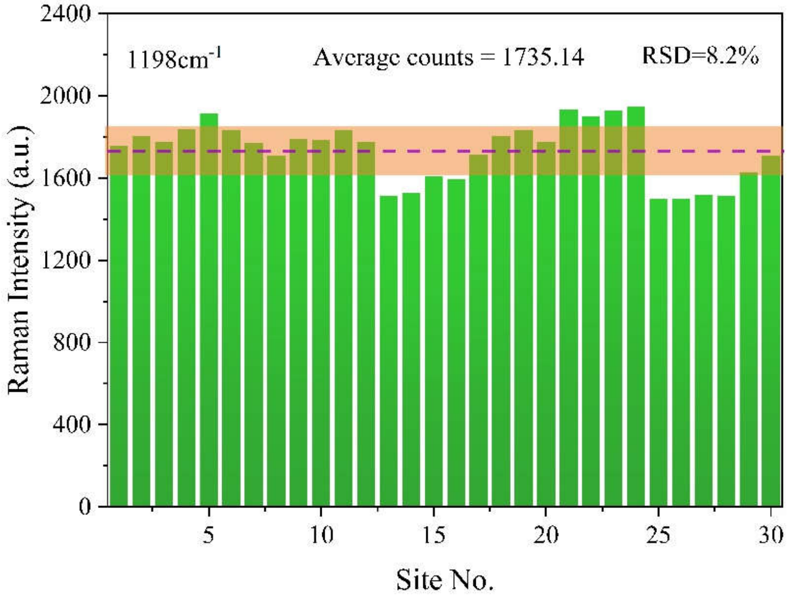

Figure S7. The signal intensity distribution at 1198 cm^{-1} for 10^{-9} M BPE recorded from 30 sites.

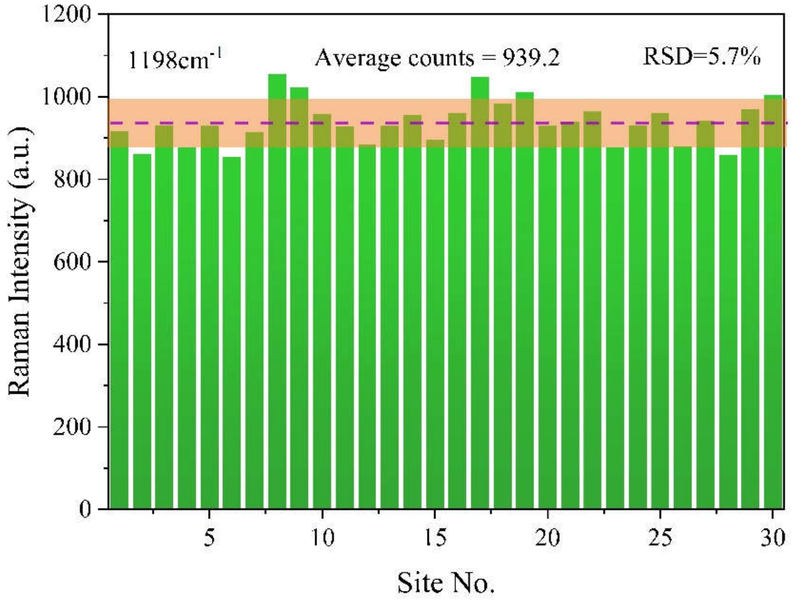

Figure S8. The signal intensity distribution at 1198 cm^{-1} for 10^{-10} M BPE recorded from 30 sites.

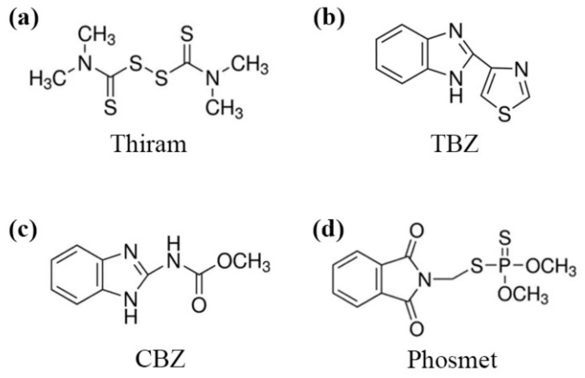

Figure S9. The chemical structures of thiram, TBZ, CBZ, and phosmet.

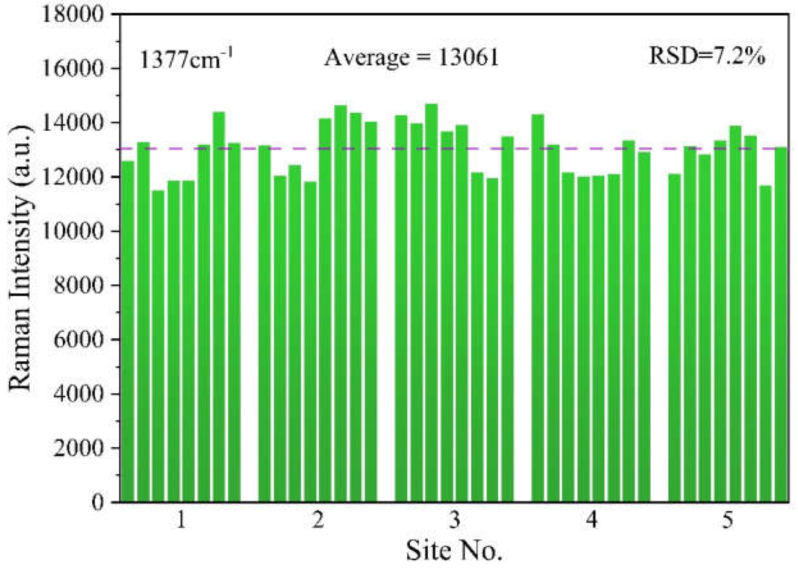

Figure S10. The signal intensity distribution at 1377 cm^{-1} for 10^{-5} M thiram. Five batches of substrates in parallel were utilized, and eight different laser spots on each substrate were chosen to yield SERS signals.

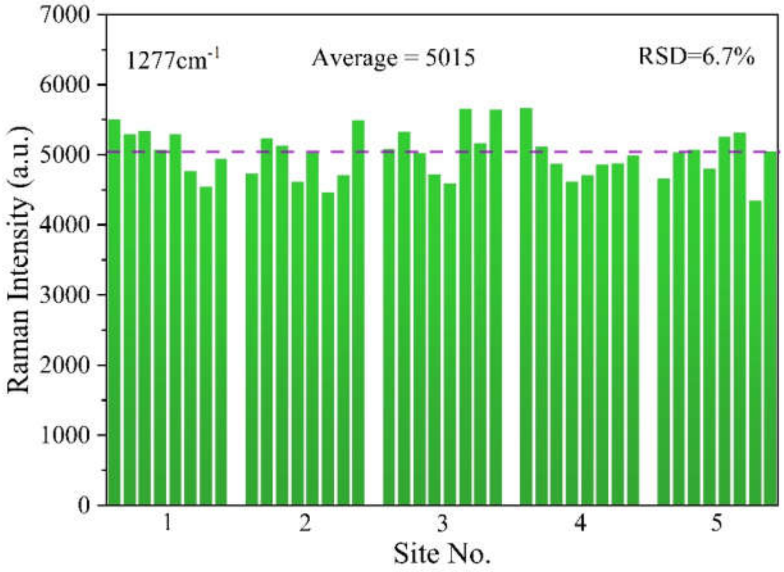

Figure S11. The signal intensity distribution at 1277 cm^{-1} for 10^{-5} M TBZ. Five batches of substrates in parallel were utilized, and eight different laser spots on each substrate were chosen to yield SERS signals.

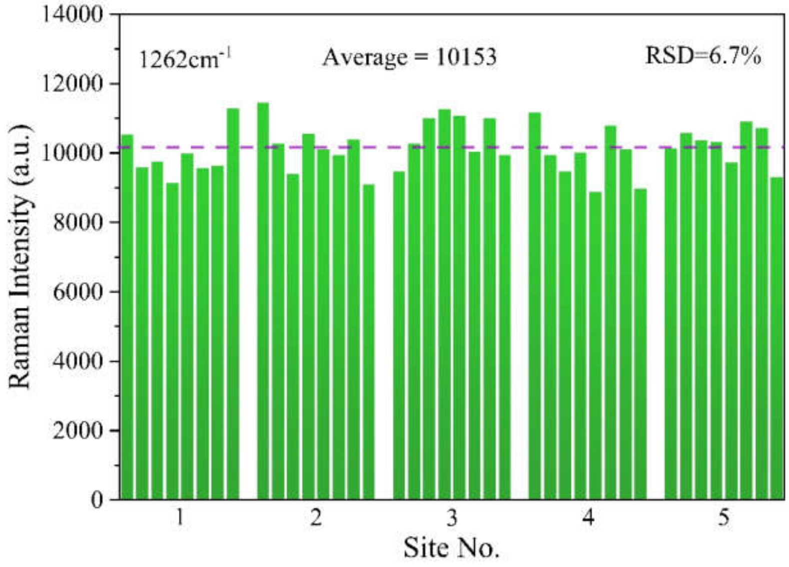

Figure S12. The signal intensity distribution at 1262 cm^{-1} for 10^{-5} M CBZ. Five batches of substrates in parallel were utilized, and eight different laser spots on each substrate were chosen to yield SERS signals.

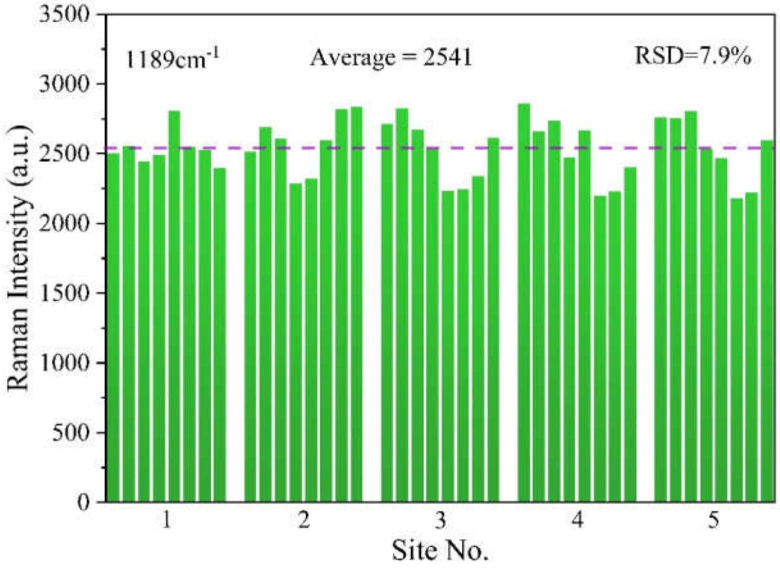

Figure S13. The signal intensity distribution at 1189 cm^{-1} for 10^{-5} M phosmet. Five batches of substrates in parallel were utilized, and eight different laser spots on each substrate were chosen to yield SERS signals.

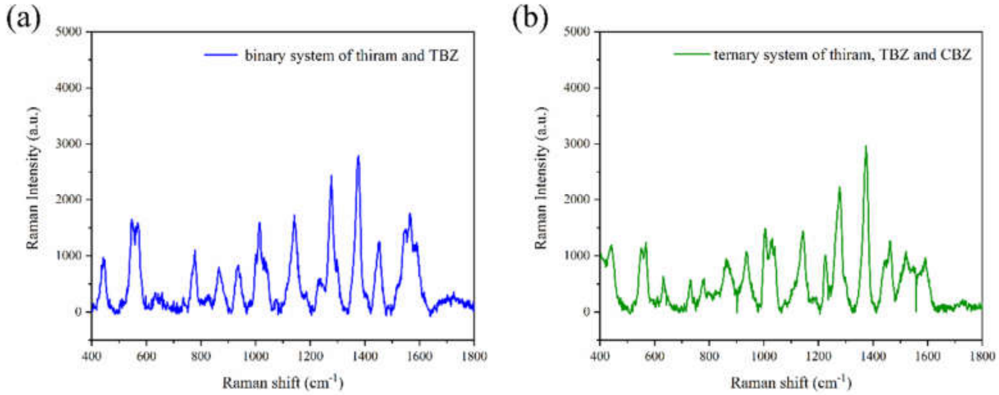

Figure S14. SERS spectrum obtained from (a) binary mixture system of thiram and TBZ; (b) ternary mixture system of thiram, TBZ and CBZ.

Table S1. Assignments of SERS bands of thiram.

SERS peaks	Assignments of SERS bands
866 cm^{-1}	stretching of C-N
935 cm^{-1}	stretching of CH3N and C=S
1043 cm^{-1}	stretching of CH3N and rocking CH3 mode
1142 cm^{-1}	stretching of C–N and rocking CH3 mode
1377 cm^{-1}	stretching of C–N and symmetric CH3 deformation
1444 cm^{-1}	antisymmetric bending CH3 deformation
1501 cm^{-1}	stretching of C–N, CH3 bending and rocking CH3 mode

Table S2. Assignments of SERS bands of TBZ.

SERS peaks	Assignments of SERS bands
780 cm^{-1}	stretching of C–H and C–N bending
875 cm^{-1}	stretching of S–C and deformation of C–C ring
1012 cm^{-1}	stretching of C–N and N=C-N bending
1277 cm^{-1}	C-H bending and ring stretching
1404 cm^{-1}	stretching of C=C
1588 cm^{-1}	stretching of C=C
1454 cm^{-1}	stretching of C=N

Table S3. Assignments of SERS bands of CBZ.

SERS peaks	Assignments of SERS bands
633 cm^{-1}	ring stretching and C=C bending
731 cm^{-1}	C–H wagging
1003 cm^{-1}	C–N bending and stretching of C–C and C–O–CH3 stretching
1028 cm^{-1}	C=N bending and stretching of C–C and stretching of C–O–CH3
1224 cm^{-1}	stretching of C–C, C–H bending and N–H bending
1262 cm^{-1}	N–H bending and C–H bending
1463 cm^{-1}	N–H bending and C–H bending
1523 cm^{-1}	N–H bending and stretching of C–N

Table S4. Assignments of SERS bands of Phosmet.

SERS peaks	Assignments of SERS bands
502 cm^{-1}	rocking vibration of CH_2 and PO_2
604 cm^{-1}	plane deformation vibrations of C=O
650 cm^{-1}	plane deformation vibrations of P=S
711 cm^{-1}	vibrations of benzene rings
1014 cm^{-1}	asymmetric stretching of P–O–C
1189 cm^{-1}	plane deformation vibrations of C–N
1775 cm^{-1}	stretching of C=O

Table S5. Identification results on the verification set.

ACC (%)	Models	Thiram
100	SVM	√
	RF	√
	KNN	√
	RF-1D-CNN	√

Table S6. Identification of thiram in mixtures of thiram and TBZ.

Mixture	Models	ACC (%)	Sensitivity (%)	Specificity (%)
Thiram + TBZ ratio: 5%:95%	SVM	77.5	55	100
	RF	55	10	100
	KNN	87.5	75	100
	RF-1D-CNN	100	100	100
Thiram + TBZ ratio: 10%:90%	SVM	82.5	65	100
	RF	100	100	100
	KNN	90	100	80
	RF-1D-CNN	100	100	100
